# Potential Roles of Bone Morphogenetic Protein (BMP)-9 in Human Liver Diseases

**DOI:** 10.3390/ijms15045199

**Published:** 2014-03-25

**Authors:** Blanca Herrera, Steven Dooley, Katja Breitkopf-Heinlein

**Affiliations:** 1 Department of Biochemistry and Molecular Biology II, Faculty of Pharmacy, Complutense University of Madrid, San Carlos Clinical Hospital Health Research Institute (IdISSC), Madrid 28040, Spain; E-Mail: bm.herrera@farm.ucm.es; 2 Department of Medicine II, Section Molecular Hepatology—Alcohol Associated Diseases, Medical Faculty Mannheim, Heidelberg University, Mannheim 68167, Germany; E-Mail: steven.dooley@medma.uni-heidelberg.de

**Keywords:** BMP-9, Hepatocellular carcinoma, signaling, physiology, liver, hepatocytes, hepatic stellate cells, angiogenesis, therapy

## Abstract

Bone morphogenetic proteins (BMP-2 to BMP-15) belong to the Transforming Growth Factor (TGF)-β superfamily and, besides their well-documented roles during embryogenesis and bone formation, some of them have recently been described to be involved in the pathogenesis of different organs, including the liver. The role of BMPs in liver damage responses including hepatocellular carcinoma (HCC) development has only begun to be addressed and strong evidence supports the concept of a pro-tumorigenic role of BMP signaling in HCC cells. BMP-9 (also termed Growth and Differentiation Factor (GDF)-2) represents the most recently discovered member of the BMP family. We have previously demonstrated that in HCC patient samples BMP-9 expression was positively associated with the tumor seize (“T stage”) and that it enhanced cell migration and induced epithelial to mesenchymal transition (EMT) in HCC cells *in vitro*. In another study we recently found that BMP-9 promotes growth in HCC cells, but not in non-transformed hepatocytes. Published as well as unpublished results obtained with primary hepatocytes support the concept of a dual function of BMP-9 in the liver: while in primary, non-malignant cells BMP-9 stabilizes the epithelial phenotype and inhibits proliferation, in HCC cells it induces cell growth and the acquisition of a migratory phenotype. In this review article we summarize current knowledge about BMPs in liver diseases, with special focus on the role of BMP-9 in HCC development and progression, that may provide new clues for a better understanding of the contribution of BMP-signaling to chronic liver diseases.

## 1. BMPs and Liver

BMPs (Bone Morphogenetic Proteins) are multifunctional cytokines that belong to the Transforming Growth Factor (TGF)-β superfamily being in fact the largest TGF-β subfamily comprising more than 15 ligands in mammals. Based on their sequence homology and function, the BMP/GDF (Growth and Differentiation Factor) subfamily can be subdivided into 7 different groups: BMP-2 and -4; BMP-5, -6, -7 and -8; GDF-5,-6 and -7; GDF-8 and -11; BMP-9 (GDF-2) and -10; GDF-1,-2 and -3 and GDF-10 and BMP-3 [1]. BMPs were originally discovered for their capacity to induce bone and cartilage formation and fracture repair and regulate growth and differentiation of chondroblast and osteoblast cells *in vitro* [2,3]. The central role of BMPs in early development is also well documented, including dorsal-ventral patterning, organogenesis and cell differentiation, and BMPs are therefore considered as potent morphogens [4,5]. In recent years, a broader role for BMPs has been unveiled and BMPs are now considered key regulators of adult tissue homeostasis [6,7]. Along these lines, numerous studies have shown that BMPs regulate a wide range of biological activities, including proliferation, differentiation, migration, chemotaxis and cell death in many different cell types. One of the target organs of BMP function that have gathered importance just recently is the liver [8]. In this regard, a well-studied aspect about the BMPs effect in liver physiology is undoubtedly their key role in the control of iron homeostasis, with BMP-6 being a master regulator of hepcidin expression. Many research groups have contributed to unravel the mechanism underlying this process which involves BMP-6 binding to its receptors and co-receptor (hemojuvenin) in hepatocytes to regulate hepcidin expression through a Smad dependent mechanism [9]. In contrast, BMP signaling, and in particular the role of BMP-7 in liver pathology is rather controversial, as both pro-fibrogenic and anti-fibrogenic activities have been demonstrated in several *in vitro* and *in vivo* models [10–17]. Bringing more complexity to this scenario, BMPs function during liver regeneration is also unclear. Thus, on one hand, BMP-7 has been shown to enhance liver regeneration after partial hepatectomy [18], but on the other hand, BMP-4 has been recently described as an inhibitor of this process [19].

Although more work is needed to clarify the exact BMP contribution to the fibrogenic and regenerative processes in the liver, all these evidences support the concept that this organ is indeed a target of BMP action, and therefore dysregulation of BMP signaling most likely has pathological consequences in the liver.

## 2. BMP-9 Signaling

**Figure 1.**
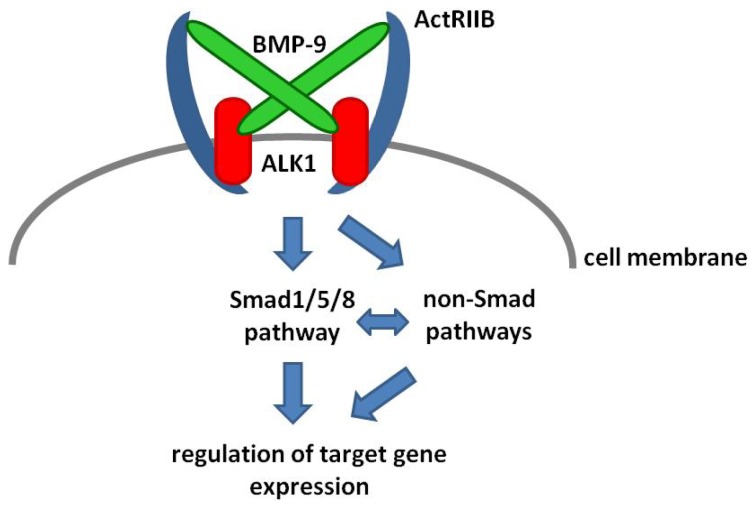
Schematic representation of the interaction of BMP-9 with its receptors. To induce down-stream Smad- or non-Smad-signaling cascades the BMP-9 dimer forms a complex with dimers of its Type I (ALK1) and type II (ActRIIB) receptors. The combination of ligand and receptors drawn in this figure represents the combination which showed the highest binding affinity but BMP-9 can also bind to other combinations of receptors (see text for details). There is no direct interaction of the extracellular domains of the type I and type II receptors and each BMP-9 monomer interacts with monomers of both types of receptors.

BMPs bind to heterotetrameric complex receptors which are localized in the cell membrane and are comprised of two different subtypes of transmembrane serine-threonine kinase receptors, termed type I (also known as Activin like kinase receptor, ALK) and type II receptors. Of the seven different type I receptors identified to date, four have been implicated in BMP signaling, namely ALK1 (ACVRL1), ALK2 (ACVRIA), ALK3 (BMPRIA) and ALK6 (BMPRIB). Three different type II receptors have been described to bind diverse BMP ligands: BMP type II receptor (BMPRII) as well as Activin type II receptor A (ActRIIA) and B (ActRIIB) (Figure 1).

Upon ligand binding receptor complexes composed of two molecules of each type I and type II receptors are formed, and the serine threonine activity of the type II receptor phosphorylates and activates the type I receptor, that in turn phosphorylates downstream signal transducing molecules. Opposite to TGF-β and activins, many BMPs bind first to the high affinity type I receptor, followed by the recruitment of type II receptors [1]. BIAcore experiments have demonstrated that BMP-9 binds with highest affinity to ALK1 and ActRIIB but with lower affinity it also binds to ALK2 and the other type II receptors BMPRII and ActRIIA [20,21]. Interestingly, recent data support the hypothesis that BMP-9 (and BMP-10) are the only ligands of the TGF-β superfamily that can bind to both type I and type II receptors with rather equally high affinity [21]. This opens the possibility for a new mechanism of “non-discriminative” formation of the heterotetrameric signaling complex. These data imply that in a cell-type with low expression of ActRIIB, BMP-9 might still signal due to its high affinity to ALK1, then forming complexes with other type II receptors. Or *vice versa*, in a cell with low levels of ALK1, the high affinity to ActRIIB might still be sufficient and signaling could be initiated via complex formation with lower affinity type I receptors (e.g., ALK2). Data from our lab and others indicate that BMP-9 could indeed signal through ALK2 when ALK1 is absent (e.g., ovarian cancer cells, C2C12 myoblasts and others) [22,23].

Some years ago, Dr. Bailly and co-workers nicely confirmed that BMP-9 and BMP-10 are the natural ligands for ALK1 in endothelial cells [24]. Indeed, BMP-9 is now recognized as key regulator of endothelial cell biology. Although expression of ALK1 was originally thought to be restricted to endothelial cells, several lines of evidence suggest that ALK1 is also expressed in different cell types, including mesenchymal cells during osteogenic differentiation, chondrocytes, cardiomyocytes and others [25].

In the liver, ALK2 and ActRIIA were found to be expressed in whole human hepatic tissue and BMPRII expression was demonstrated in primary mouse hepatocytes and HCC cell lines [26,27]. Initial studies had already shown that BMP-9 binds specific receptors in the HCC cell line HepG2 [28], and further data indicated that these cells express ALK2, BMPRII, ActRIIA and ActRIIB [27,29,30], presenting all the components required for BMP-9 signaling. Interestingly, our results indicate that the human, non-differentiated hepatoma cell line, HLE also expresses ALK1, which contributes to BMP-9 signaling in these cells [29]. It will be interesting to analyze whether ALK1, ActRIIB or other BMP-9 receptor expression is gained in HCC cells, the mechanism how this occurs and the specific contribution to the hepatocarcinogenic process.

**Figure 2.**
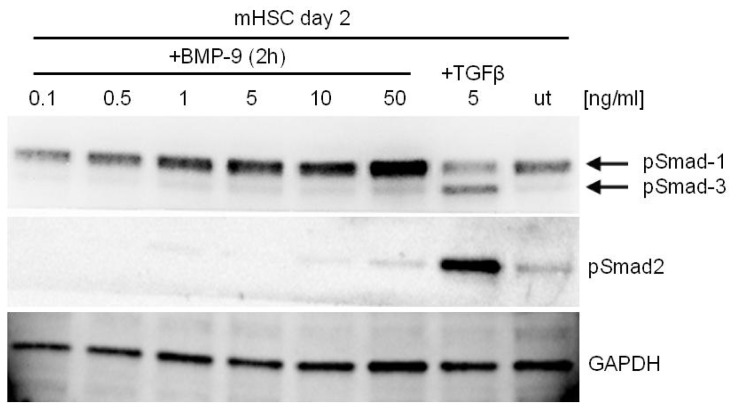
BMP-9 induces Smad-signalling in mouse hepatic stellate cells (mHSC). HSC were isolated from healthy mouse livers and after 2 days of primary culture, the cells were stimulated with BMP-9 for 2 h. Stimulation with TGF-β for 2 h was used as positive control for activation of the Smad-2/3 pathway. Phosphorylated Smad proteins were detected by Western blot [32] and GAPDH levels were determined as loading control. While BMP-9 dose-dependently led to phosphorylation of Smad-1 only, TGF-β, as expected, led to activation of the Smad-2/3 pathway. ut = untreated.

Besides hepatocytes, non-parenchymal cells seem also responsive to BMP-9: Hepatic stellate cells (HSC) express ALK1, and its levels remain unchanged in transdifferentiating cells, although BMP-9 signaling and function has not yet been studied in these cells [31]. Ongoing experiments from our lab show that HSC are indeed responsive to BMP-9 in terms of Smad-1 phosphorylation (Figure 2).

In the same line, Miller *et al.* also demonstrated that BMP-9 binds to specific receptors in liver endothelial cells and Kupffer cells [28]. At the time these experiments were conducted, the nature of these receptors was unclear but with the present knowledge it is possible to hypothesize that the liver endothelial cell receptor was indeed ALK1. Similarly, macrophages have been shown to express ALK1 [33], therefore it is reasonable to consider that the BMP-9 binding receptor in Kupffer cells is in fact also ALK1, although direct evidence is yet lacking.

## 3. Co-Receptors and Extracellular Regulators

BMP signaling is modulated by the presence of co-receptors and extracellular regulators. Among the co-receptors, endoglin seems to be of special relevance, although the precise nature of this relation is not well understood. It has been nicely demonstrated that BMP-9 binds with high affinity to endoglin [23,34]. Some data suggest that siRNA mediated knockdown of endoglin did not alter BMP-9 signaling in endothelial cells [35], and even, it has been postulated that endoglin inhibits BMP-9 interaction with type II receptor, thereby blocking BMP-9 signaling [34]. However, most of the data in the literature indicate that endoglin enhances BMP-9 signaling, being therefore required for maximal phosphorylation of Smad-1 [24,36,37]. In the liver, endoglin is found to be expressed in liver sinusoidal endothelial cells and hepatic stellate cells. In fact, endoglin expression is up-regulated during activation of these cells *in vitro*. Endoglin expression is also enhanced during experimental liver fibrosis, and serum endoglin levels have been associated with hepatic fibrogenesis [13,38]. Another important piece of data is the fact that, although hepatocytes do not express endoglin, transformed human HCC cells do [13]. How endoglin interacts with BMP-9 in the liver scenario remains to be delineated, yet this line of research appears to be fairly exciting. Other co-receptors comprehended as the Repulsive Guidance Molecule Family do enhance BMP ligand affinity for its receptors. Unlike other BMPs BMP-9 does not bind to hemojuvelin or any other of the members of this family [39]. An additional level of BMP signaling regulation is achieved by extracellular regulators like soluble, secreted proteins that associate with BMP ligands and therefore impair their binding to their receptors. In fact the different networks formed by the Dan, twisted gastrulation and noggin and chordin families are central in the control of dynamic morphogenetic fields of BMP activity during development [40]. Recently it has been elegantly demonstrated that crossveinless2 (CV2), a member of the chordin family, binds to BMP-9 and inhibits its activity. The authors further showed that BMP-9 induces CV2 expression in endothelial cells. CV2, in a negative feedback loop, binds with high affinity to BMP-9 and thereby inhibits its binding to ALK1 and limits its own activity [41]. On the other hand, noggin is unable to bind BMP-9 and to inhibit BMP-9 osteogenic activity in mesenchymal cells, and indeed recent data have enlightened the structural reasons behind the BMP-9 resistance to noggin mediated inhibition [42–44]. Clearly, extensive efforts are required to deeper understand the BMP-9 network of receptors, co-receptors and extracellular regulators that operate in the liver.

## 4. BMP-9—Smad Dependent Signaling

Like other BMPs, BMP-9 binding to its receptors triggers the phosphorylation of the R-Smads, Smad1,5,8. The activation of this pathway has been documented in all cellular types analyzed up to date, including hepatocytes and HCC cells regardless of the receptors utilized by BMP-9 [29,45]. Of note, some authors additionally have described that BMP-9 triggers Smad-2/Smad-3 phosphorylation, which is classically associated with TGF-β signaling. Thus, Upton and co-workers described that BMP-9 stimulated Smad2 but not Smad3 phosphorylation in different endothelial cells, and furthermore Smad2 appeared to be involved in gene regulation induced by this cytokine [35]. Others reported Smad2 and Smad3 phosphorylation induced by BMP-9 in different endothelial cell types [23,46]. Although phosphorylation of Smad2 in response to BMP-9 could not be detected in HepG2 cells (unpublished observations, Herrera B., Complutense University of Madrid, Madrid, Spain, 2013), primary hepatocytes (unpublished observations, Breitkopf-Heinlein K., Heidelberg University, Mannheim, Germany, 2014) or mouse HSC (Figure 2) BMP-9 mediated activation of Smad2 and/or Smad3 in other hepatic cellular types remains to be determined.

## 5. BMP-9—Non-Smad Dependent Signaling

In certain cellular models, BMPs activate Smad independent pathways, referred to as non-canonical or non-Smad pathways that include MAPK (p38, ERK and JNK), PI3K/AKT, NF-κB, Wnt, Rho-GTPase and modulation of microRNAs. Hence, BMPs have been shown to induce Smad and non-Smad signaling pathways simultaneously and/or sequentially which enables them to modulate different biological functions [8,47]. Mechanisms involved in the activation of non-Smad pathways are not completely understood, but type I receptor association with TAK1 (TGF-β activated kinase 1), TAB1 (TGF-β activated kinase 1/MAP3K7 binding protein 1), and XIAP (X-linked inhibitor of apoptosis) have been associated with MAPKs activation [47]. Little is known about BMP-9 induced non-canonical pathways and most of the data available in the literature have been obtained in mesenchymal progenitor cells (MPC). In this model, BMP-9-mediated-JNK activation is involved in osteogenic differentiation of MPCs [48]. The authors also found that BMP-9 triggers p38 and ERK activation that, in turn, appears to modulate the Smad pathway: p38 inhibition decreases Smad1,5,8 phosphorylation induced by BMP-9 whereas ERK inhibition results in the opposite effect. Accordingly, these MAPKs seem to play opposing roles in BMP-9 induced osteogenic differentiation in MPCs. Thus, BMP-9-triggered p38 activation mediates MPC proliferation and also early and late osteogenic differentiation. In contrast, ERK inhibition results in an increase of BMP-9-mediated osteogenic markers of differentiation [49,50]. Whether the effects observed in BMP-9-mediated osteogenic differentiation by ERK and p38 inhibition are the result of Smad signaling modulation or a direct effect of the MAPKs on this process is not known. Along these lines, p38 activation induced by BMP-9 has been documented in other cell types such as osteosarcoma cells [51], human osteoclasts derived from cord blood monocytes [52] and dental follicle stem cells [53]. In the former case, together with p38 activation, downregulation of phospho-ERK was achieved upon BMP-9 treatment. In lung microvascular endothelial cells, BMP-9 activates both p38 and ERK, although the biological function mediated by these MAPKs was not identified [46]. There are no reports of BMP-9 induced non-Smad pathways in liver cells. Yet, our unpublished data indicate that p38 and AKT are both activated by BMP-9 in HepG2 cells and p38 seems to be required for the survival effect of this cytokine (unpublished work. García-Álvaro *et al.*, Complutense University of Madrid, Madrid, Spain, 2014).

## 6. BMP-9 Mediated Regulation of Target Gene Expression

First evidence for BMP-9 mediated gene regulation comes from studies performed in human multipotent mesenchymal cells. In this model, BMP-9 induced Sox9, a transcription factor that regulates expression of two markers of chondrogenic differentiation, Col2A1 and aggrecan [54]. Extensive work has been carried out analyzing the BMP-9-mediated osteogenic differentiation in mesenchymal progenitor cells (MPC) that led to the identification of a set of target genes that may be involved in this process. In seminal studies using Affymetrix GeneChip expression analysis, Dr He’s group found more than 40 genes regulated by BMP-9 in MPC, including classical BMP targets such as the inhibitor of DNA binding transcription factors Id1, Id2 and Id3, and the inhibitory Smads, Smad6 and Smad7 [55,56]. They also found differential expression of other genes whose relevance in the context of osteogenic differentiation induced by BMP-9 remains to be elucidated (e.g., FOXO4, calpain II and matrix metalloproteinase11). The transcription factor Hey1 which belongs to the basic helix-loop-helix HERP family, was also induced by BMP-9 in a Smad dependent manner, and data convincingly show its involvement in BMP-9 mediated osteogenic differentiation [56]. Smad dependent expression of Cox2 was also found in the former expression profile analysis and Cox2 mediates BMP-9-induced osteogenic differentiation by regulating the expression of osteopontin and osteocalcin. Interestingly, Cox2 seems to directly modulate Smad1,5,8 signaling and Smad dependent gene expression in MPC [57]. Along these lines, the transcription factor Runx2, which is central for osteoblast differentiation by directly stimulating the expression of most of the well established bone markers (among them alkaline phosphatase and osteopontin), has been shown to be a target gene of BMP-9 in several cellular models like MPCs, osteosarcoma and dedifferentiated fat cells [51,56,58]. It is worth mentioning that the hypoxia inducible factor, Hif1α, has been recently described as a target of BMP-9 in MSC. Hence BMP-9 mediated Hif1α up-regulation is dependent on the Smad pathway and contributes to BMP-9 induced osteogenic differentiation [59]. Further work is necessary to extend these observations to other cell types, and it will be most interesting to analyze the contribution of BMP-9 mediated regulation of Hif1α in cancer processes. The key role of BMP-9 in the induction and maintenance of the cholinergic phenotype of basal forebrain cholinergic neurons has been known for some time and it does so by modulating a specific collection of genes. In a profound analysis performed in septal cells using Affimetrix arrays, it was shown that BMP-9 regulated several proteins related to neuronal differentiation and acquisition of a cholinergic phenotype, such as choline acetyl transferase and the neurotrophin receptor. It also modulates a number of genes involved in cell-cell interaction (e.g., Cadherin 6 and 11); extracellular matrix components (Col9a1, Col9a3 and Col1a1); cell cycle control (cyclin dependent kinase inhibitor 1C) and BMP signaling (Alk3, noggin and Roaz, a transcription factor involved in DNA-binding-Smad1 complexes). Importantly, BMP-9 also modulates the expression of nerve growth factor (NGF), both at the mRNA and protein levels, that is involved in the induction and maintenance of the cholinergic phenotype of septal cells. Furthermore, BMP-9 regulates neurotrophin3, Igf1 and noggin in this system which suggests that BMP-9 could function by modulating the trophic environment of developing neurons [60,61]. It is well established that BMP-9 up-regulates Id1, Id2, Id3, Smad6, Smad7, endoglin and BMPRII in HUVEC cells and other endothelial cell types [23,24,35,62] and recent studies have revealed that BMP-9 modulates several genes that are crucial for endothelial cell biology. EphrinB2 is a tyrosine kinase receptor, expressed in arterial endothelial cells, that together with its ligand EphB4 defines arteries and veins, therefore preventing arterio-venous anastomosis. Using different endothelial cell models, the authors show that BMP-9 up-regulates EphrinB2 expression, by a mechanism dependent on ALK1/Id1-Id3 cooperation. BMP-9 also induces the transcription factor Hey1 that regulates vascular endothelial growth factor receptor (VEGFR) expression, thereby decreasing endothelial cell responsiveness to VEGF [63]. Endothelin (ET)-1 is a peptide with vasoconstrictor and mitogenic properties, which has been associated with pulmonary arterial hypertension. BMP-9 induces ET-1 expression in different endothelial cell models, although the intracellular mechanism of this regulation is controversial as p38 involvement in this process has been documented in some cellular models, but not in others. Nevertheless, data are suggestive of a key role of ET-1 in proliferation and tubule formation by BMP-9 in human artery endothelial cells [46,64]. TMEM100 is a recently described BMP-9 regulated gene that encodes for an intracellular transmembrane protein involved in arterial endothelium differentiation and vascular morphogenesis. Human umbilical artery endothelial cells induce TMEM100 in response to BMP-9 in a Smad1 dependent manner, and importantly TMEM100 deficient mice present vascular abnormalities resembling those found in the ALK1 knockout mouse [62]. ALK1 expression has also been documented in lymphatic endothelial cells and these cells respond to BMP-9 by inducing Smad6 expression. BMP-9 seems to have an important function in the formation of intraluminal lymphatic valves that are required for directional flow of the lymphatic system. Data reveal that BMP-9 regulates master genes controlling vasculogenesis, like forkhead box C2 and Cx37 together with other genes such as EprinB2 and neuropilin 1 [65].

In the liver, the best known target of gene regulation by BMP-9 is hepcidin. Hepcidin is a small peptide produced by hepatocytes that controls the iron metabolism: hepcidin induces degradation of ferroportin, an iron exporter, thus preventing iron absorption. Hepcidin expression is modulated by different factors, including systemic iron, oxygen levels, inflammation, oxidative stress and, importantly, by BMPs. In fact, hepcidin transcription regulation is dependent on the Smad pathway [66] and its promoter contains two identical and highly conserved BMP responsive elements [67]. Further investigation has defined BMP-6 as an essential regulator of hepcidin expression, by binding to BMP receptors and the co-receptor hemojuvelin in hepatocytes [68]. Nevertheless, hepcidin was also shown to be induced by several other BMP ligands *in vitro*, including BMP-9, that indeed appeared to be remarkably potent [69], although BMP-9 does not functionally interact with hemojuvelin [70–73]. In line with these observations, BMP-6 knockout mice, which present an iron overload phenotype, display increased expression of other BMPs, including BMP-9, in the small intestine, but this compensatory up-regulation was not sufficient to balance BMP-6 absence [26].

**Figure 3.**
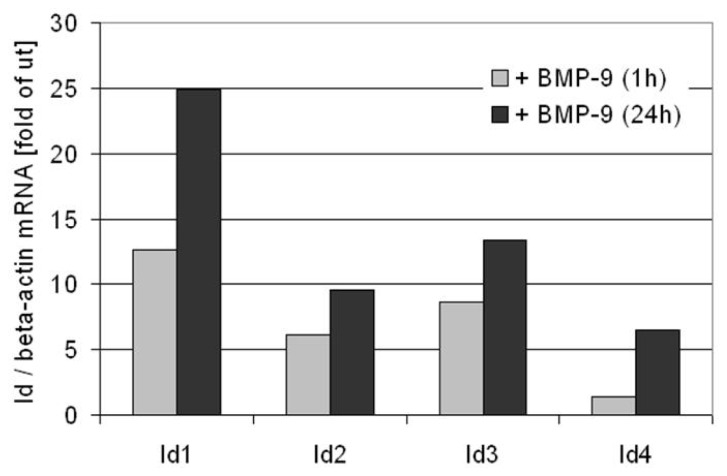
BMP-9 induces expression of Id-proteins in primary cultured mouse hepatocytes. Hepatocytes were isolated from healthy mouse livers and after 24 h of primary culture, the cells were stimulated with BMP-9 (5 ng/mL) for 1 or 24 h as indicated under serum-free culture conditions. Expression of all four known isoforms of *inhibitor of differentiation* (*Id*) was determined by micro array analysis (Affymetrix, High Wycombe, UK; hybridization to MOE430_2 Affymetrix GeneChips (Santa Clara, CA, USA)). The bars represent the average values for *Id* mRNA expressions normalized to corresponding *β-actin* values of two independent measurements with RNA pooled from four different cell isolations. ut = untreated.

But hepcidin is not the only BMP-9-target gene described in liver. We have shown that Id1 is strongly up-regulated in HepG2 cells [45] as well as primary hepatocytes (Figure 3) in response to BMP-9 and the EMT-master regulator Snail is found to be induced in HCC cells. As the analysis of this transcription factor expression level was performed after 72 h of BMP-9 treatment, an indirect effect cannot be ruled out, and indeed, the molecular mechanism of Snail induction by BMP-9 remains to be elucidated [29]. By using reporter assay approaches with constructs containing promoters of different glucose and lipid metabolism related enzymes, Chen *et al.* described that BMP-9 mediated profound effects at this level resembling the insulin effect. Thus, BMP-9 was shown to regulate different proteins involved in hepatic fatty acid metabolism: BMP-9 increases malic enzyme, fatty acid synthase and sterol regulatory element binding protein (SREBP-1c) reporters activity. The authors also found that BMP-9 inhibits phosphoenolpyruvate carboxykinase transcription [74]. Since all these data were generated in H4IIe, a rat hepatoma cell line, it would be of interest to analyze whether these transcriptional effects can also be found in both untransformed and transformed human hepatocytes. In summary, the current data support the notion that BMP-9 gene regulation is highly dependent on the target cell. Nevertheless, a set of genes appear to be commonly regulated in most of the cellular models analyzed to date, including, the Id transcription factors.

## 7. BMP-9 *versus* BMP-10

Many important signaling pathways rely on multiple ligands. It is unclear if this is a mechanism of safeguard via redundancy or if it serves other functional purposes. Among TGF-β family members, BMP-9 and BMP-10 share the highest sequence homology and both are physiological ligands for ALK1 [24]. ALK1 inactivation in mice leads to embryonic lethality at E11 because of major angiogenesis defects supporting the important role of BMP-9 and BMP-10 for angiogenesis [75,76]. But are their functions in general fully overlapping? The answer is no. BMP-10 for example, has an exclusive function in cardiac development, which cannot be substituted by BMP-9 [77]. In this work the authors provide genetic evidence that defines two distinct functions of BMP-10, one, which can be functionally substituted by BMP-9, that supports vascular development via ALK1-dependent signaling in endothelial cells, and another one which regulates heart development in a BMP-10-exclusive manner.

Bmp-9^−/−^ mice were born at the expected Mendelian ratios, developed normally, and grew into fertile adults [77]. It seems that this mainly normal development of the knock-out animals is a result of BMP-9 being fully replaced by BMP-10 during embryogenesis. Nevertheless, Bmp-9-knockout neonates and adult mice had decreased lymphatic draining efficiency. These data identify BMP-9 as an important extracellular regulator in the maturation of the lymphatic vascular network affecting valve development and lymphatic vessel function [65]. Obviously the normal expression of BMP-10 could not compensate for this defect. Considering the fact that lymphatic vessels may also serve as a conduit to lymph nodes BMP-9 might thereby also participate in mediating systemic metastasis of cancer cells. Ricard *et al*. further showed that Bmp-9 deficiency did not alter postnatal blood vascularization of the mouse retina, whereas addition of a neutralizing anti-BMP-10 antibody in these pups had a dramatic effect, demonstrating that BMP-10 could compensate for the loss of BMP-9 in retinal angiogenesis [78]. As described above, among the three type II receptors BMPRII, ActRIIA and ActRIIB implicated in BMP-9 and BMP-10 signaling, BMP-9 exhibits striking preference towards ActRIIB while BMP-10 binds to all type II receptors with rather equal affinities. This might be one explanation why there is no complete redundancy. Thus despite apparent sequence homology and possibly similar functions BMP-9 and BMP-10 could act through different type II receptors [21].

Another possible explanation for BMP-9 *versus* BMP-10 specific actions may be that active BMP-10 might not be available within a certain tissue type. Most BMPs are required to become locally activated by proteases of the furin/subtilisin pro-protein convertase family [79]. BMP-9 being available in the circulation already in a biologically active form is efficient as is, while BMP-10 circulates in an inactive form needing local activation to become efficient. Therefore one could speculate that the local activation machinery for BMP-10 might not always be present within a given environment. Another possibility is that circulating BMP-10, although being unable to activate BRE activity (a Smad1/5 reporter), could still be able to signal via Smad-independent mechanisms.

Addition of serum to endothelial cells induced a phospho-Smad1/5 response that could be completely inhibited by the addition of a neutralizing anti-BMP-9 antibody, supporting the concept that in the adult BMP-9 plays a major role while BMP-10 function seems mainly to be restricted to embryogenesis [44,80]. Nevertheless a possible redundancy of the functions of these two BMPs should still be kept in mind when planning to apply BMP-9 neutralizing strategies for future therapies in patients.

## 8. BMP-9: Role in Liver Physiology

We already know that BMP-9 is eminently expressed in the liver [28,80]. In fact, BMP-9 was first isolated from fetal mouse liver [81] and later Miller *et al.* described it to be expressed in rat liver, specifically in non-parenchymal cells [28]. In contrast, in recent studies performed in mouse and human liver BMP-9 mRNA was not detected in liver endothelial cells, nor in hepatic stellate cells, but rather in hepatocytes and biliary epithelial cells [80]. Along these lines, we have also shown that BMP-9 is detectable in normal human liver by immunohistochemistry [29,45]. It is plausible that BMP-9 expression would be different across species; nevertheless, a more systematic analysis of mRNA and protein levels of this cytokine in the different liver cell types is mandatory. Little is known about the regulation of BMP-9 expression in the liver. The only data available come from studies performed in the Dr. Bordin’s laboratory: glucose and insulin combination triggered an increase of BMP-9 expression in rat perfused livers. Together with these *ex vivo* experiments, *in vivo* assays revealed that oral glucose administration in overnight fasted rats also increased BMP-9 levels in liver and furthermore both mRNA and protein levels were diminished in different models of insulin resistance [82]. Taken together, these evidences point to insulin and glucose as potential regulators of BMP-9 expression in liver, nevertheless more work is needed to fully clarify the mechanisms involved. On the other hand, the identification of other regulators of BMP-9 expression is of great interest; in particular, as documented for other TGF-β family members, a BMP-9 auto-regulatory feedback loop is plausible. Importantly, BMP-9 serum levels change during development [80]. As liver is the major source of BMP-9 in the organism, one could speculate that BMP-9 expression in the liver also varies during development. If that is the case, BMP-9 levels would be modulated by liver development itself and key master regulators would be worthy to determine. It is necessary, however, to consider that liver cells would be able to respond not only to BMP-9 present in the hepatic tissue in an autocrine/paracrine manner, but also these cells are exposed to existing serous BMP-9 since it is present in mouse and human serum at bioactive concentrations [44,80,83].

Most of the knowledge we have nowadays about BMP-9 action in liver parenchymal cells has been obtained in transformed hepatocytes, and will be discussed next. In normal, non-transformed hepatocytes, we have shown that BMP-9 triggers phosphorylation of Smad1,5,8. We were also able to show that BMP-9 incubation moderately decreases cell viability of adult mouse hepatocytes in primary culture, whereas it did not have any effect in the non-tumoral immortalized human hepatocyte cell line THLE3 nor in SV40 immortalized mouse neonatal hepatocytes [45]. In contrast, in primary rat hepatocytes, the opposite effect had been documented, with BMP-9 being a mild inducer of cell proliferation [81]. Although these latter findings are in contrast to our own observation that BMP-9 reduces basal and HGF-mediated proliferation of primary mouse hepatocytes *in vitro* (unpublished work, Breitkopf-Heinlein K., Heidelberg University, Mannheim, Germany, 2014). These discrepancies could be explained by the different origin of the hepatocytes (human, rat or mouse) as well as by the experimental approaches utilized. Yet, if BMP-9 exerts further functions in the normal liver physiology remains to be fully clarified. In fact, a couple of studies suggest a role for BMP-9 in the (liver) glucose homeostasis [74,82]. Although *in vitro* data present some caveats already mentioned, *in vivo* experiments are rather compelling: recombinant BMP-9 injection decreases glycemic levels in normal and diabetic mice, with slower kinetic than insulin. The data suggest that this impressive effect is dependent on BMP-9 mediated insulin release by pancreatic beta cells by an indirect and yet unknown mechanism, and also with a direct action of BMP-9 in the transcription regulation of fatty acid and glucose metabolism related enzymes in the liver [74]. Accordingly neutralization of BMP-9 with an anti-BMP-9 antibody resulted in glucose intolerance and insulin resistance in fasted rats [82]. Further studies are required to fully confirm the exciting hypothesis that BMP-9 is a hypoglycemic factor as potent as insulin; particularly, it would be essential to elucidate the molecular mechanism underlying this BMP-9 effect in the liver context.

## 9. BMP-9: Role in Neo-Angiogenesis

Angiogenesis is a process which is of high importance during embryonic development but is mainly quiescent at the adult stage. However, angiogenesis is re-activated during processes of wound repair and under several pathological conditions, such as tumor growth and metastasis (see below).

BMP’s are by now well recognized as important modulators of angiogenesis [84]. An important point in understanding how BMPs regulate angiogenesis is to know their receptor localization. ALK2, ALK3, ALK6 and BMPRII are expressed in almost all cells, while the expression of ALK1 and endoglin is mostly restricted to endothelial cells.

One well characterized vascular disease related to mutations in the BMP signaling system is the Rendu-Osler-Weber syndrome also known as hemorrhagic hereditary telangiectasia (HHT). HHT is an autosomal dominant vascular disorder, which presents with mucosal and skin telangiectasia, pulmonary, cerebral and hepatic malformations, and hemorrhages associated with these vascular lesions. Mutations in three different genes have been causally linked to HHT: (1) The ENG gene encoding the co-receptor endoglin causing HHT1 [85]; (2) the ACVRL1 gene encoding ALK1 causing HHT2 [86]; and (3) the SMAD4 gene causing a mixed syndrome consisting of both juvenile polyposis and HHT [87].

Important pro-angiogenic factors include VEGF and basic fibroblast growth factor (FGF2), which stimulate proliferation and migration of endothelial cells. Some BMPs were described to directly induce VEGF-A: BMP-7 in primary cultures of fetal rat calvaria cells and BMP-2, BMP-4 and BMP-6 in osteoblasts and preosteoblast-like cell lines [88,89]. However, BMP-9 and BMP-10 seem to play rather inhibiting roles concerning neo-angiogenesis, at least in normal, non-malignant tissues/cells. It was shown that BMP-9 inhibited neo-angiogenesis *in vivo*, in the mouse sponge assay and inhibited blood circulation in the CAM-assay [44] while BMP-10 was reported to inhibit proliferation and migration of dermal HMVECs [24]. As mentioned above, BMP-9 is present in serum and plasma at a concentration of around 5 ng/mL [44,80,83] thereby BMP-9 is the only BMP circulating at a higher concentration than its EC50 (50 pg/mL for ALK1). In addition, BMP-9 is not inhibited by the circulating antagonist noggin [44]. Based on these findings it can be concluded that in the adult organism BMP-9 is the major endothelial-stimulating BMP and that it is responsible for basal Smad1/5/8 phosphorylation in endothelial cells. Considering its general inhibitory effects on angiogenesis, BMP-9 was proposed as a major circulating vascular quiescence factor [44]. Along with this concept, one hypothesis for explaining the etiology of HHT is that a deficient BMP9/ALK1/endoglin pathway might lead to re-activation of angiogenesis leading to endothelial hyperproliferation and therefore vasodilation, endothelial cell hypermigration and arteriovenous malformation [84].

In sharp contrast to this view of BMP-9 as anti-angiogenic factor, Suzuki *et al.* found that BMP-9 enhances proliferation of three other kinds of endothelial cells: mouse embryonic-stem-cell-derived endothelial cells (MESECs); mouse normal endothelial cells and tumor associated endothelial cells (TECs). Since the same group on the other hand confirmed an anti-proliferative effect on human dermal microvascular endothelial cells (HMVECs) it seems that the outcome of BMP-9 on proliferation strongly depends on the type of endothelial cell which is targeted [90]. Suzuki *et al.* also reported that BMP-9 enhanced the angiogenesis in *ex vivo* culture of allantois and in *in vivo* Matrigel plug assay. Whether BMP-9 acts pro- or anti-angiogenic in the diseased liver and how its’ anti-proliferative and migratory effects are turned around during tumorigenesis (see below) remains to be investigated.

## 10. Roles of BMP-9 in HCC and Other Types of Cancer

The molecular mechanisms that underlie new blood vessel development in tumors have been an area of intense study which has led to the development of several therapeutics like bevacizumab (an antiVEGF antibody) or multireceptor tyrosine kinase inhibitors such as sunitinib, sorafenib, and imatinib [91], all aiming at limiting the formation of new blood vessels within tumors by neutralizing or inhibiting the action of angiogenic factors. Novel targets for anti-angiogenic therapy could provide treatment alternatives in patients whose tumors are unresponsive to approved agents such as VEGF pathway inhibitors. The BMP-9/BMP-10/ALK1 pathway is a promising target for anti-angiogenic cancer therapy and with dalantercept (previously known as ACE-041) a new drug is now being tested, displaying promising antitumor activity in patients with advanced refractory cancer of diverse origins including liver [92], and multiple phase II studies will follow soon. Dalantercept is a soluble receptor fusion protein consisting of the extracellular domain of human ALK1 linked to the Fc portion of human IgG1. Besides dalantercept, an anti-ALK1 antibody (Pfizer, New York, NY, USA) is undergoing development as a potential antitumor agent [93,94].

As described above, endoglin (CD105), a transmembrane protein and co-receptor for members of the transforming growth factor superfamily, also plays a crucial role in angiogenesis and has been shown to bind to BMP-9 and -10. Since endoglin, like ALK1, binds to BMP-9 and -10, a soluble form of this protein, coupled to an Fc-domain was used to neutralize BMP-9 in the colon-26 mouse tumor model and, similar to soluble ALK1, it reduced the tumor burden in these mice due to its anti-angiogenesis properties [34].

Using the RIP1-Tag2 mouse model of endocrine pancreatic tumorigenesis [95], Cunha *et al.* reported an increased expression of BMP-9 and TGF-β within the developing tumor. By crossing the RIP1-Tag2 mice with ALK1 +/− mice they further demonstrated that genetically reduced ALK1 expression retards tumor progression through the angiogenic switch, reduces *de novo* tumor growth, and impairs angiogenesis in this model of pancreatic islet carcinoma [96]. Interestingly, together with reduced ALK1 expression tumors of these mice also showed reduced expression of ALK5 and endoglin, implying some kind of cross-talk between BMP-9 and TGF-β signaling pathways. Inhibition of ALK1 signal transduction using a truncated form of the receptor coupled to an Fc-domain led to decreased angiogenesis in diverse *in vitro* models (e.g., in a spheroid sprouting assay using endothelial cells, HUVEC) as well as decreased tumor development in the RIP1-Tag2 mouse model [96]. As mentioned above, TGF-β first binds to the type-II receptor and subsequently recruits the type-I receptor, this order is reversed for some members of the BMP family [1]. Therefore this soluble form of ALK1 only neutralizes BMP-9 (and -10) but not TGF-β or other BMPs. These results imply that the neo-angiogenic properties of BMP-9 are of pivotal relevance for tumor formation *in vivo*. Importantly, HCC is a hypervascularized tumor [97] and ALK1 expression in liver tumor blood vessels has been shown to be high [93], facts that make HCC a good candidate for ALK1 inhibition therapeutic strategy. With regard to these findings it remains to be shown if the effects of soluble ALK1 are indeed due to neutralization of BMP-9. Would reduced expression or knock-out of BMP-9 itself directly result in the same reduced angiogenesis and tumorigenesis as described above for ALK1-Fc?

**Figure 4.**
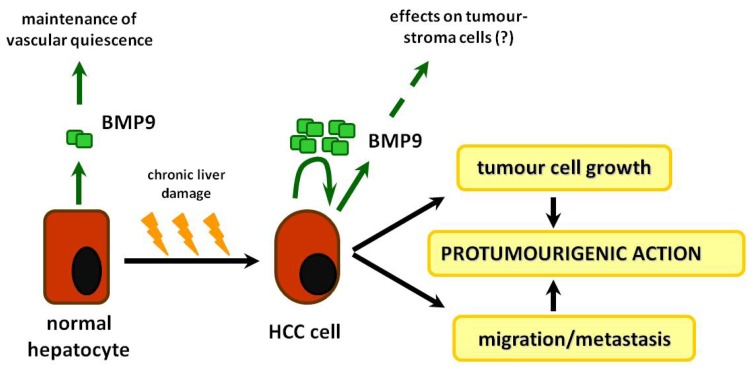
Schematic representation of BMP-9 effects on hepatocytes. Relatively high amounts of BMP-9 are already present in the healthy liver where it helps to stabilize existing vessels and the epithelial phenotype of hepatocytes. Upon chronic damage hepatocytes can undergo malignant transformation and especially cells at the tumor border then produce high amounts of BMP-9 which down-regulate the cell-cell contacts of peri-tumoral cells, induce epithelial to mesenchymal transition (EMT) of the tumor cells and thereby promote metastasis formation. How BMP-9 might act on peri-tumoral stroma cells (fibroblasts) or other cell types surrounding the tumor remains to be investigated. In contrast to normal hepatocytes HCC cells respond to BMP-9 with enhanced proliferation.

Furthermore, in other types of cancer like the epithelial ovarian cancer [22] and especially in the liver, BMP-9 seems to exert more effects than modulating neo-angiogenesis. We have recently shown that in human HCC tissue samples BMP-9 levels positively correlate with metastasis formation and with markers of epithelial-to-mesenchymal transition (EMT) like induction of Snail and reduction of E-Cadherin [29]. Accordingly BMP-9 induced EMT-like changes in phenotype of the HCC cell lines HepG2 and HLE *in vitro*. In line with these findings Maegdefrau and Bosserhoff reported that general inhibition of BMP-signaling using dorsomorphin, noggin or chordin reduced the migratory and invasive behavior of HCC cells [98]. BMP-9 has also proliferative and anti-apoptotic effects in HCC cells: our *in vivo* and *in vitro* data suggest that BMP-9 production is increased in a subset of HCC, and that this autocrine loop enhances cell growth. Collectively, all these data point to a pro-tumorigenic role of BMP-9 by enhancing tumor cell growth and metastasis formation in HCC (Figure 4).

## 11. Summary, Conclusions and Outlook

In summary, the available data point to BMP-9 being an important cytokine for maintenance of normal liver function as well as mediating cell-type specific hepatic wound-healing responses that may lead to fibrosis/cirrhosis and finally HCC. BMP-9 seems to differ significantly from other BMPs in terms of receptor-selectivity and its sensitivity to signaling regulators/inhibitors. Further investigations of the complex interplay of the BMP-9 responses of the different liver cell types and their inter-cellular cross-talk in normal physiology and disease is urgently needed. Such knowledge will most likely lead to promising new therapeutic strategies for treatment of liver diseases including HCC.

## Abbreviations

BMPs: Bone morphogenetic proteins
TGF: Transforming growth factor
HCC: Hepatocellular carcinoma
GDF: Growth and differentiation factor
EMT: Epithelial to mesenchymal transition
ALK: Activin like kinase
ActRIIA: Activin type II receptor A
ActRIIB: Activin type II receptor B
HSCs: Hepatic stellate cells
Smad: a portmanteau of “mothers against decapentaplegic” (Drosophila homologue) and the protein “SMA” (Caenorhabditis elegans homologue)
GAPDH: Glyceraldehyde-3-phosphate dehydrogenase
CV2: Crossveinless2
PI3K: Phosphatidylinositol-3-kinase
Akt: Protein kinase B
JNK: c-Jun *N*-terminal kinase
Erk: Extracellular signal regulated kinase
NF-κB: Nuclear factor kappa B
Wnt: Wingless (Drosophila homologue)
TAK 1: TGF-β activated kinase 1
TAB1: TGF-β activated kinase 1/MAP3K7 binding protein 1
XIAP: X-linked inhibitor of apoptosis
MAPK: Mitogen activated protein kinase
MPC: Mesenchymal progenitor cell
ColxA1/2: Collagen type x, alpha-1 or -2 chain
Id: Inhibitor of differentiation
Hif1α: hypoxia inducible factor 1α
NGF: Nerve growth factor
IGF: Insulin like growth factor
VEGF: Vascular endothelial growth factor receptor
ET: Endothelin
TMEM100: Transmembrane protein 100
SREBP: Sterol regulatory element binding protein
BMPRII: BMP receptor type II
ActRIIA/B: Activin receptor type IIA/B
BRE: BMP response element
SV40: Simian virus 40
HHT: Hemorrhagic hereditary telangiectasia
ENG: Endoglin
FGF: fibroblast growth factor
CAM: Chick chorioallantoic membrane
MESECs: Embryonic-stem-cell-derived endothelial cells
TECs: Tumor associated endothelial cells
HMVECs: Human dermal microvascular endothelial cells
